# Drug Interaction between Sirolimus and Ranolazine in a Kidney Transplant Patient

**DOI:** 10.1155/2014/548243

**Published:** 2014-01-02

**Authors:** Joanna C. Masters, Mita M. Shah, Ashley A. Feist

**Affiliations:** ^1^Skaggs School of Pharmacy and Pharmaceutical Sciences, University of California, San Diego, La Jolla, CA 92093, USA; ^2^University of California, San Diego Medical Center, Center for Transplantation, La Jolla, CA 92037, USA; ^3^University of California, San Diego Medical Center, Department of Medicine, Division of Nephrology and Hypertension, San Diego, CA 92103, USA

## Abstract

*Purpose*. The case of a kidney transplant recipient who experienced a probable drug interaction between sirolimus and ranolazine is reported. *Summary*. The narrow therapeutic window of immunosuppressive therapy in transplant recipients requires close monitoring for potential drug-drug interactions. The patient, a 57-year-old Caucasian male kidney transplant recipient, was stable for years on sirolimus as his primary immunosuppressive agent and had a history of chronic angina, for which he was prescribed ranolazine. Upon addition and dose escalation of ranolazine, whole blood sirolimus levels more than tripled, rising to immeasurably high concentrations. After holding sirolimus on multiple occasions and reducing dosage more than 50%, blood levels returned to therapeutic range, while continuing ranolazine. *Conclusion*. Since ranolazine is a documented P-GP and CYP3A inhibitor, and sirolimus a known substrate for both pathways, it is proposed that ranolazine inhibition of P-GP and CYP3A4 contributed to the significant elevation in sirolimus exposure. No alternative causes for the rise in sirolimus exposure were found, and assessment with the Drug Interaction Probability Scale finds this interaction to be probable. Clinicians should be aware of the potential for this interaction to cause elevated sirolimus exposure and subsequent increase in clinical effect or toxicity, in this case overimmunosuppression.

## 1. Introduction

Immunosuppressive agents used in solid organ transplant have narrow therapeutic windows, making drug-drug interactions a major concern. As new drugs are brought to market, clinically significant drug interactions may be identified that have not been previously reported in the literature. This case report describes a possible drug interaction between sirolimus and ranolazine in a kidney transplant patient, leading to supratherapeutic blood concentrations of sirolimus.

Ranolazine is a piperazine derivative approved for treatment of refractory angina; however, its exact mechanism of action is unclear. Ranolazine is metabolized primarily by intestinal and liver enzymes CYP3A (cytochrome P450, family 3A), and to a lesser extent CYP2D6, resulting in active metabolites, with less than 5% of compounds excreted in the urine. It is reported to be a mild inhibitor of CYP3A. Ranolazine is also a substrate and moderate inhibitor of p-glycoprotein (P-GP), an active transporter that serves as an intestinal efflux pump [1, 2].

The immunosuppressant sirolimus is a mammalian target of rapamycin (mTOR) inhibitor, which is used in transplant patients in place of, or along with, calcineurin inhibitors such as cyclosporine and tacrolimus. Sirolimus is metabolized primarily by CYP3A4 and about 2% is excreted in urine. Sirolimus is also a substrate and mild inhibitor of the P-GP efflux pump [3]. Due to the narrow therapeutic window, drug interactions through CYP or P-GP mechanisms are of concern, and increased knowledge about such interactions can prevent unnecessary toxic accumulation of sirolimus and significant adverse events.

The purpose of this paper is to report a probable drug interaction between sirolimus and ranolazine, leading to elevations in sirolimus blood levels. To further assess this interaction, an advanced literature search of the PubMed database was performed, using the search terms ranolazine and sirolimus. No results were reported. This lack of published information regarding concomitant use of these two agents highlights the importance of presenting this case report.

## 2. Case Report

A 57-year-old Caucasian male presented to the long-term kidney transplant clinic for routine follow-up. The patient's medical history was significant for type 1 diabetes mellitus since the age of ten leading to complications of coronary artery disease with chronic angina and end-stage renal disease. Four years prior to presentation, the patient received a simultaneous pancreas and kidney transplant. The patient underwent pancreatectomy shortly after transplantation due to graft thrombosis, but the kidney allograft continued to function. Additionally, he underwent a redo coronary artery bypass graft (CABG) one year after transplant. Due to an intolerance of mycophenolate mofetil and tremor on therapeutic-dose tacrolimus, he has been maintained on an immunosuppressive regimen of sirolimus, low-dose tacrolimus, and prednisone since shortly after transplantation. His renal function has been stable with a serum creatinine ranging from 1.3 to 1.7 mg/dL.

**Figure 1:**
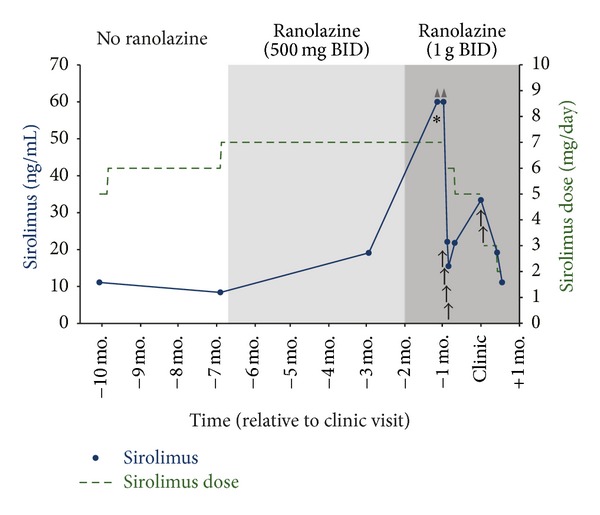
Timeline of sirolimus dosing and whole blood concentrations with ranolazine titration. ▲: level above limit of detection; **↑**: sirolimus held for 1 day; ∗: patient presents to emergency department.

The patient had remained adherent and stable on sirolimus for several years with whole blood levels of 8–12 ng/mL on doses of 5 to 7 mg by mouth daily. Approximately seven months prior to presentation to our transplant clinic, the sirolimus dose was increased from 6 mg to 7 mg daily. One week later, the patient presented to his cardiologist with worsening angina and was started on ranolazine 500 mg by mouth twice daily (Figure 1). Four months after the addition of ranolazine, sirolimus trough concentration was measured to be 19.1 ng/mL, higher than previous values, but still within the target therapeutic range. No changes were made to his immunosuppressive regimen at this time. Due to ongoing angina, shortly thereafter the ranolazine dose was increased twofold to 1 gram twice daily. No other significant changes in his medication profile were made throughout this time period.

One month following this ranolazine dose increase, the patient presented to the emergency department for pain, dehydration, and malaise following a recent oral surgery. In the emergency department, the sirolimus level was found to be greater than 60 ng/mL, which is beyond the upper limit of detection of the assay. A repeat level confirmed this value. After consultation with the transplant nephrology service, the sirolimus was held for four days, until concentrations reached 15.5 ng/mL. All other laboratory values were within normal limits, including renal and hepatic function tests. Sirolimus was then restarted at a decreased dose of 6 mg daily; however, repeat levels were once again above the therapeutic target, and therefore the dose was further reduced one week later to 5 mg daily (see Figure 1 for detailed timeline). At this time, the etiology of the elevated sirolimus levels was unclear and had not been associated with a possible drug-drug interaction.

In following month the patient presented for follow-up to our kidney transplant clinic. His medication list at presentation included sirolimus 5 mg daily with a target trough concentration of 10–20 ng/mL, low-dose tacrolimus 1 mg daily, prednisone 5 mg daily, clopidogrel 75 mg daily, aspirin 325 mg daily, ranolazine 1 gram twice daily, amlodipine besylate 10 mg daily, lisinopril 10 mg daily, metoprolol tartrate 100 mg twice daily, furosemide 20 mg twice daily, nitroglycerin sublingual spray 0.4 mg as needed for chest pain, hydromorphone hydrochloride 4 mg every 4 hours as needed for pain, vitamin D 5,000 IU daily, and insulin lispro administered via insulin pump. The patient reported taking all medications as prescribed, and the only major medication changes in the recent past were titration of ranolazine and sirolimus as described previously. At this visit his steady-state sirolimus trough level was elevated to 33.4 ng/mL. Close review of his recent sirolimus levels and dosing history was made by the clinical pharmacist and transplant nephrologist, and an association between the addition of ranolazine and the highly elevated sirolimus levels was identified. A drug interaction was suspected between ranolazine and sirolimus, based on ranolazine's moderate P-GP inhibition and mild CYP3A inhibition. The patient was instructed to hold two doses of sirolimus, and reduce his daily dose to 3 mg thereafter with close monitoring of his sirolimus levels. He would continue on ranolazine 1 gram twice daily as instructed by his cardiologist and was advised to notify the transplant clinic of any future changes in ranolazine therapy.

The patient returned for labs in two weeks, at which time his sirolimus level was 19.2 ng/mL. He was then instructed to completely discontinue his low-dose tacrolimus due to the risk of potential overimmunosuppression. Six months later his sirolimus levels have remained within the target range on 2-3 mg daily, and he has continued on the same dose of ranolazine 1 gram twice daily.

## 3. Discussion

Ranolazine is reported to be a moderate inhibitor of P-GP, and the manufacturer includes a caution that dose reduction may be needed in agents transported by P-GP when used in combination with ranolazine. Ranolazine is also a CYP3A inhibitor, and package information contains a generic caution for use with drugs with narrow therapeutic windows, including sirolimus, tacrolimus, and cyclosporine. However, no published reports were found on the specific drug interaction or magnitude of interaction between sirolimus and ranolazine. Two published case reports describe an interaction between tacrolimus and ranolazine in transplant recipients, proposing that both the weak CYP3A inhibition and the moderate P-GP inhibition were responsible for observed elevated tacrolimus levels, which in one case required a 70% decrease in tacrolimus dose [4, 5]. Another case report suggests that the same dual P-GP and CYP3A inhibition was responsible for increased simvastatin exposure upon addition of ranolazine, which resulted in toxicity of rhabdomyolysis [6]. Ranolazine concentrations were not measured for this patient during the reported time period, and therefore systemic ranolazine levels cannot be assessed in this case.

Of note, this patient was also concurrently on tacrolimus. However, he was on a low dose given once daily with levels below the limit of detection of the assay and had no changes in tacrolimus dose during the time period detailed in this report. After the addition of ranolazine, tacrolimus levels increased to the detectable range but still remained subtherapeutic, therefore requiring no dose adjustment. For this reason, the drug interaction between ranolazine and tacrolimus, which has been reported previously [4, 5], was not a primary focus of this case report.

Drugs such as ranolazine that have both CYP and P-GP inhibition have the potential to create large increases in systemic concentrations of agents that are substrates of both, such as sirolimus. An increase in bioavailability through inhibited P-GP efflux, in addition to a decrease in first-pass metabolism and systemic clearance through inhibition of CYP enzymes, may result in sirolimus blood levels several-fold above the target systemic concentration. Such an event was described in a patient whose sirolimus levels increased more than eightfold upon addition of clarithromycin, a potent inhibitor of both P-GP and CYP3A [7]. Sirolimus blood levels far above the therapeutic window can have dire consequences for the patient, including complications of overimmunosuppression leading to serious infections. Patients on immunosuppressants such as sirolimus and concomitant dual CYP and P-GP inhibitors require closer monitoring of systemic drug levels, particularly after any relevant medication and/or dose changes. Patients should be clearly instructed to report any changes instituted by other providers to the transplant clinic team for appropriate follow-up, including up-to-date drug levels and increased monitoring, if necessary.

Alternative explanations do exist for the phenomenon described here; however, upon review we have determined none to be highly probable in this patient case. Of course, we assume that the patient was adherent to the prescribed sirolimus regimen, as this is what he reported during discussion with providers and there was no evidence of changes in prescription refill patterns that would suggest overdosing. The presence of other concomitant medications resulting in a drug interaction with sirolimus and/or ranolazine would also contribute to supratherapeutic sirolimus exposure. We could identify no other alterations in medication regimen with the potential to significantly impact ranolazine or sirolimus exposure. The patient's hepatic function remained normal throughout this time, making acute hepatic impairment an unlikely explanation for impaired sirolimus clearance. High fat meals have been shown to increase the mean total exposure (AUC) of sirolimus by 23 to 35% [3]; however, an unreported change in dosing time with regard to meals or any dietary changes is also not likely to explain the magnitude and the duration of change in sirolimus level seen in this case.

There is also a possibility that the degree of the P-GP interaction between ranolazine and sirolimus and subsequent increase in sirolimus bioavailability is impacted by dosing times. Simultaneous administration of sirolimus and modified cyclosporine, a known CYP3A4 and P-GP inhibitor, has been shown to increase the mean maximum concentration (*C*_max_) of sirolimus by 512% and AUC by 148% compared with administration of sirolimus alone. However, administering sirolimus four hours after modified cyclosporine, as directed by the package insert, results in only a 33% increase in both *C*_max_ and AUC [3]. Perhaps a similar phenomenon could be observed with ranolazine and sirolimus, and thus minimizing the amount of ranolazine present in the gut to inhibit P-GP efflux could reduce the extent of increase in sirolimus exposure.

## 4. Conclusion

In this case we observed that upon the addition of ranolazine 1 gram twice daily, sirolimus dosing had to eventually be reduced by greater than fifty percent. Based on the sequence of events and known mechanisms listed above, we believe that the doubling of the daily ranolazine dose was the precipitating factor in the significant elevation of sirolimus trough levels. The phenomenon observed here did not represent an acute event, but a sustained interaction that persisted while the patient continued on ranolazine. No reasonable alternative causes for the magnitude of increase in sirolimus exposure could be found. The combination of inhibition of P-GP efflux and CYP3A4 enzymatic clearance by ranolazine explains the mechanism that may have caused the great increase in sirolimus concentration that occurred shortly thereafter.

This is the first published report of a drug interaction between ranolazine and sirolimus resulting in elevated sirolimus exposure. The patient was able to continue treatment on both sirolimus and ranolazine once doses were appropriately adjusted based on frequent blood level measurements. We determined the patient experienced a probable drug interaction through evaluation with the Drug Interaction Probability Scale (DIPS). This scale is a tool which aids in objective assessment of causation in patient cases involving suspected drug interactions [8]. Further study is needed to improve understanding of the mechanism and extent of the drug interaction, as well as methods to reduce the severity, such as dose separation. Transplant recipients taking sirolimus should be instructed to report any medication changes to the providers managing their transplant medications and take care to monitor for any signs of infection or other new adverse effects. Furthermore, other providers should be encouraged to check for any potential interactions with narrow therapeutic range immunosuppressants before adding or removing medications for comorbid conditions.
